# Tris(3,5-di­aza-1-azonia-7-phosphaadamant-7-yl)(1,3,5-tri­aza-7-phospha-adamantane)silver(I) dicarbonate

**DOI:** 10.1107/S2414314626007935

**Published:** 2026-08-14

**Authors:** Sizwe J. Zamisa, Adesola A. Adeleke, Olatunde S. Olatunji, Bernard Omondi

**Affiliations:** a School of Agriculture and Science, Discipline of Chemistry, University of KwaZulu-Natal, Private Bag X54001, Durban, 4000, South Africa; University of Aberdeen, United Kingdom

**Keywords:** crystal structure, neutral 1,3,5-tri­aza-7-phosphaadamantane (PTA), monoprotonated PTAH, carbonate ion, silver ion

## Abstract

The title compound, [Ag(C_6_H_13_N_3_P)_4_(C_6_H_12_N_3_P)](CO_3_)_2_ or [Ag(PTAH)_3_(PTA)](CO_3_)_2_, is a discrete cationic silver(I) complex composed of one neutral 1,3,5-tri­aza-7-phosphaadamantane (C_6_H_12_N_3_P; PTA) ligand and three monoprotonated PTAH^+^ (C_6_H_13_N_3_P^+^) ligands coordinated to the silver center, resulting in an overall +4 charge balanced by two carbonate anions. The compound crystallizes in the rhombohedral *R*3 space group with the silver ion and PTA P atom lying on a crystallographic threefold axis, as does one of the carbonate C atoms. The silver ion adopts a tetra­hedral geometry with P—Ag—P bond angles near the ideal 109°, and silver–phospho­rus bond distances of 2.4699 (6) and 2.4683 (11) Å for Ag—P_PTA_ and Ag—P_PTAH_, respectively. The crystal packing features N—H⋯O hydrogen bonds between the protonated N atoms of the PTAH ligands and the non-coordinating carbonate anions.

## Abstract 2

In the title salt, the silver(I) ion is coordinated by both neutral 1,3,5-tri­aza-7-phosphaadamantane (PTA) and protonated PTAH^+^ ligands.

## Graphical abstract

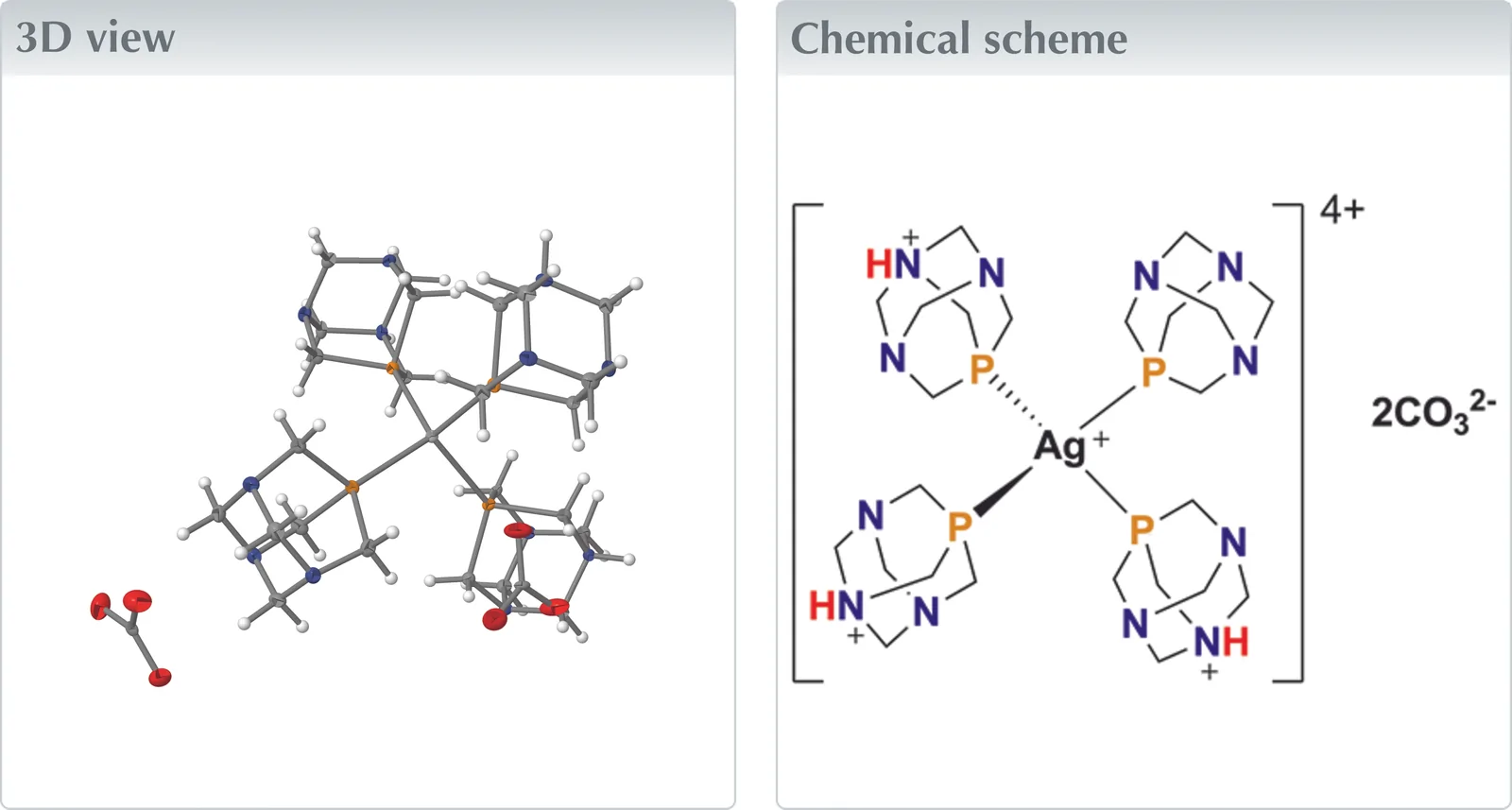

## Structure description

The phosphine ligand 1,3,5-tri­aza-7-phosphaadamantane (C_6_H_12_N_3_P; PTA) has garnered growing inter­est because of its water solubility and distinctive coordination properties arising from a soft phospho­rus donor and three nitro­gen atoms (Krogstad *et al.*, 2007 ▸). While PTA mainly binds to metals *via* the phospho­rus atom, its nitro­gen atoms can enable diverse coordination modes, impacting complex stability and functionality (Darensbourg *et al.*, 1997 ▸). Silver(I) complexes with PTA display enhanced solubility and stability useful in catalysis and biomedical fields (Jimenez *et al.*, 2017 ▸; Armstrong *et al.*, 2018 ▸).

As part of our studies in this area, this work reports the synthesis and crystal structure of the title salt [Ag(PTAH)_3_(PTA)](CO_3_)_2_ (**I**), illustrating the coordination of silver(I) with both neutral PTA and protonated PTAH ligands in the presence of carbonate counter-ions.

**Figure 1:**
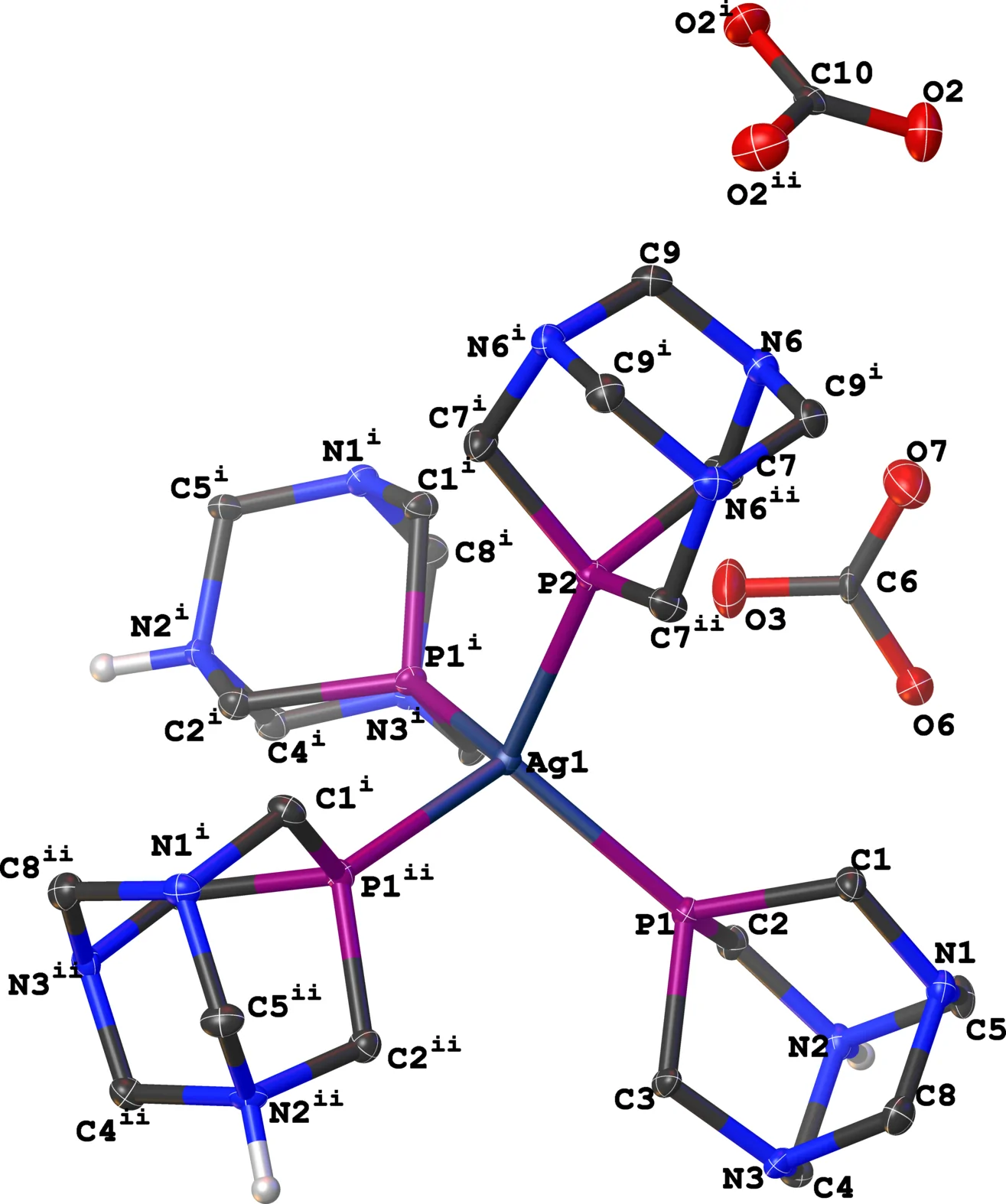
The mol­ecular structure of (**I**) shown with displacement ellipsoids at the 50% probability level. All C-bound hydrogen atoms are omitted for clarity. Symmetry codes: (i) 1 − *y*, *x* − *y*, *z*; (ii) 1 + *y* − *x*, 1 − *x*, *z*.

**Figure 2:**
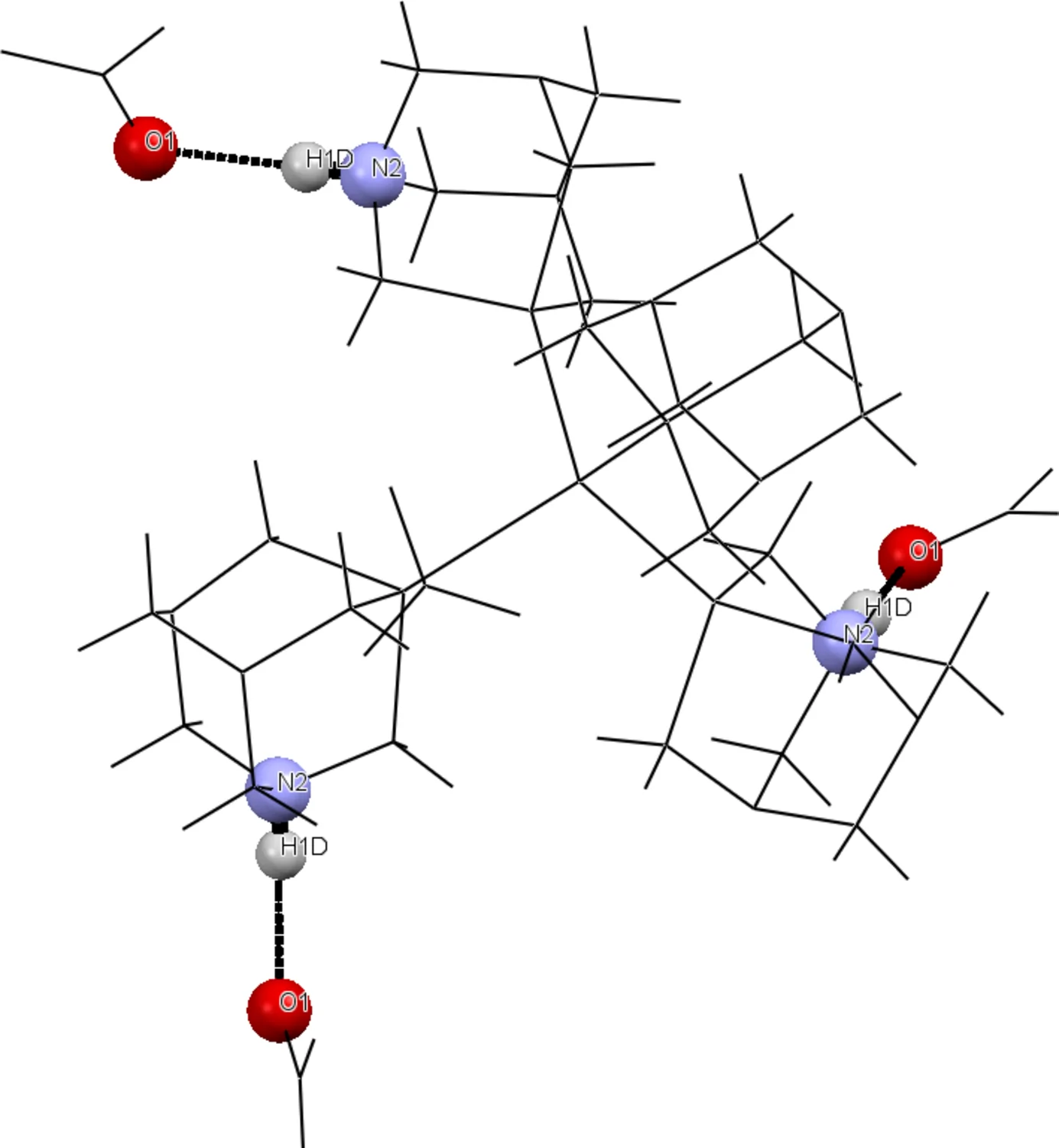
Representation of N—H⋯O hydrogen bonds in the crystal structure of (**I**) (blue and red dotted bonds).

The asymmetric unit of (**I**) consists of an Ag ion (site symmetry 3), a complete PTAH ligand, a fragment of PTA (P site symmetry 3), a complete carbonate anion and a fragment of a carbonate ion (C site symmetry 3), as shown in Fig. 1 ▸. The complete cation is constructed through symmetry code 1 − *y*, *x* − *y*, *z*, which results in the silver atom coordinated with one neutral PTA and three monoprotonated PTA ligands, forming a cationic complex with a +4 charge balanced by two carbonate anions. The Ag center again exhibits tetra­hedral geometry, with bond angles averaging close to the ideal tetra­hedral angle of 109°. The Ag—P1 and Ag—P2 bond distances are 2.4699 (6) and 2.4683 (11) Å, respectively. The P—Ag—P bond angles are comparable to those of other tetra-coordinated metal phosphine complexes such as [Cu(PTA)_4_][BF_4_]·6H_2_O and [Cu(PTAH)_4_][NO_3_]_5_ with an average P—Cu—P bond angle of 109.5° (Kirillov *et al.*, 2007 ▸, Porchia *et al.*, 2009 ▸). In the extended structure of (**I**), the carbonate ions accept strong N—H⋯O hydrogen bonds arising from the protonated nitro­gen atoms of the HPTA ligands (Fig. 2 ▸). These hydrogen bonds are characterized by an N⋯O distance of 2.731 (3) Å and an N—H⋯O angle of 176°, indicating strong and directional inter­molecular inter­actions (Table 1 ▸).

## Synthesis and crystallization

Formic acid (0.3 ml, 8.0 mmol) was added slowly to silver(I) oxide (0.93 g, 4.0 mmol) in water (10 ml), generating silver(I) formate, which was filtered to remove unreacted material. PTA (1.26 g, 8.0 mmol) dissolved in acetone (20 ml) was added dropwise to the filtrate, yielding a cloudy solution. Slow evaporation of the filtered solution afforded X-ray quality crystals of (**I**) suitable for analysis. The carbonate ions in the product may have arisen from CO_2_ absorbed from the atmos­phere in the presence of silver ions (Barnes *et al.*, 1971 ▸; Kong *et al.*, 2005 ▸).

## Refinement

Crystal data, data collection and structure refinement details are summarized in Table 2 ▸.

## Supplementary Material

Crystal structure: contains datablock(s) I. DOI: 10.1107/S2414314626007935/hb4568sup1.cif

Structure factors: contains datablock(s) I. DOI: 10.1107/S2414314626007935/hb4568Isup2.hkl

CCDC reference: 2578472

Additional supporting information:  crystallographic information; 3D view; checkCIF report

## Figures and Tables

**Table 1:** Hydrogen-bond geometry (Å, °)

*D*—H⋯*A*	*D*—H	H⋯*A*	*D*⋯*A*	*D*—H⋯*A*
N2—H2⋯O6^i^	1.00	1.73	2.731 (3)	176

Symmetry code: (i) 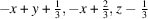.

**Table 2:** Experimental details

Crystal data	
Chemical formula	[Ag(C_6_H_13_N_3_P)_4_(C_6_H_12_N_3_P)](CO_3_)_2_
*M*_r_	859.52
Crystal system, space group	Trigonal, *R*3
Temperature (K)	173
*a*, *c* (Å)	15.7992 (12), 13.1502 (18)
*V* (Å^3^)	2842.7 (6)
*Z*	3
Radiation type	Mo *K*α
μ (mm^−1^)	0.78
Crystal size (mm)	0.36 × 0.24 × 0.22

Data collection	
Diffractometer	Bruker APEXII CCD
Absorption correction	Multi-scan (*SADABS*; Krause *et al.*, 2015 ▸)
*T*_min_, *T*_max_	0.767, 0.847
No. of measured, independent and observed [*I* > 2σ(*I*)] reflections	18709, 3005, 2981
*R*_int_	0.022
(sin θ/λ)_max_ (Å^−1^)	0.668

Refinement	
*R*[*F*^2^ > 2σ(*F*^2^)], *wR*(*F*^2^), *S*	0.019, 0.048, 1.08
No. of reflections	3005
No. of parameters	172
No. of restraints	13
H-atom treatment	H-atom parameters constrained
Δρ_max_, Δρ_min_ (e Å^−3^)	0.77, −0.32
Absolute structure	Flack *x* determined using 1393 quotients [(*I*^+^)−(*I*^−^)]/[(*I*^+^)+(*I*^−^)] (Parsons et al., 2013 ▸)
Absolute structure parameter	−0.013 (5)

Computer programs: *APEX2* and *SAINT* (Bruker, 2014 ▸), *SHELXS97* (Sheldrick, 2008 ▸), *SHELXL2018/3* (Sheldrick, 2015 ▸) and *OLEX2* (Dolomanov *et al.*, 2009 ▸).

## full crystallographic data

### (I) Crystal data

**Table:**

[Ag(C_6_H_13_N_3_P)_4_(C_6_H_12_N_3_P)](CO_3_)_2_	*D*_x_ = 1.506 Mg m^−^^3^
*M**_r_* = 859.52	Mo *K*α radiation, λ = 0.71073 Å
Trigonal, *R*3	Cell parameters from 9969 reflections
*a* = 15.7992 (12) Å	θ = 2.6–28.3°
*c* = 13.1502 (18) Å	µ = 0.78 mm^−^^1^
*V* = 2842.7 (6) Å^3^	*T* = 173 K
*Z* = 3	Block, colourless
*F*(000) = 1518	0.36 × 0.24 × 0.22 mm

### (I) Data collection

**Table:**

Bruker APEXII CCD diffractometer	2981 reflections with *I* > 2σ(*I*)
Graphite monochromator	*R*_int_ = 0.022
φ and ω scans	θ_max_ = 28.3°, θ_min_ = 2.2°
Absorption correction: multi-scan (SADABS; Krause *et al.*, 2015)	*h* = −21→17
*T*_min_ = 0.767, *T*_max_ = 0.847	*k* = −15→21
18709 measured reflections	*l* = −17→17
3005 independent reflections	

### (I) Refinement

**Table:**

Refinement on *F*^2^	Hydrogen site location: inferred from neighbouring sites
Least-squares matrix: full	H-atom parameters constrained
*R*[*F*^2^ > 2σ(*F*^2^)] = 0.019	*w* = 1/[σ^2^(*F*_o_^2^) + (0.031*P*)^2^ + 1.7576*P*] where *P* = (*F*_o_^2^ + 2*F*_c_^2^)/3
*wR*(*F*^2^) = 0.048	(Δ/σ)_max_ = 0.001
*S* = 1.08	Δρ_max_ = 0.77 e Å^−^^3^
3005 reflections	Δρ_min_ = −0.32 e Å^−^^3^
172 parameters	Absolute structure: Flack *x* determined using 1393 quotients [(*I*^+^)-(*I*^-^)]/[(*I*^+^)+(*I*^-^)] (Parsons et al., 2013)
13 restraints	Absolute structure parameter: −0.013 (5)
Primary atom site location: dual	

### (I) Special details

**Table:**

Geometry. All esds (except the esd in the dihedral angle between two l.s. planes) are estimated using the full covariance matrix. The cell esds are taken into account individually in the estimation of esds in distances, angles and torsion angles; correlations between esds in cell parameters are only used when they are defined by crystal symmetry. An approximate (isotropic) treatment of cell esds is used for estimating esds involving l.s. planes.
Refinement. Refinement of F^2^ against ALL reflections. The weighted R-factor wR and goodness of fit S are based on F^2^, conventional R-factors R are based on F, with F set to zero for negative F^2^. The threshold expression of F^2^ > 2sigma(F^2^) is used only for calculating R-factors(gt) etc. and is not relevant to the choice of reflections for refinement. R-factors based on F^2^ are statistically about twice as large as those based on F, and R- factors based on ALL data will be even larger.

### (I) Fractional atomic coordinates and isotropic or equivalent isotropic displacement parameters (Å^2^)

**Table:**

	*x*	*y*	*z*	*U*_iso_*/*U*_eq_	
C7	0.54971 (19)	0.2863 (2)	0.48563 (19)	0.0157 (5)	
H7A	0.518427	0.324777	0.465991	0.019*	
H7B	0.505181	0.217503	0.465855	0.019*	
C8	0.5965 (2)	0.6287 (2)	0.1338 (2)	0.0154 (5)	
H8A	0.657144	0.668832	0.173223	0.018*	
H8B	0.573099	0.672593	0.108986	0.018*	
C9	0.6073 (2)	0.2336 (2)	0.6302 (2)	0.0157 (5)	
H9A	0.605558	0.230630	0.705424	0.019*	
H9B	0.567006	0.166064	0.604429	0.019*	
N6	0.56354 (17)	0.29178 (17)	0.59648 (17)	0.0146 (4)	
C1	0.55921 (19)	0.4978 (2)	0.25461 (19)	0.0134 (5)	
H1A	0.508041	0.450520	0.301041	0.016*	
H1B	0.616570	0.542448	0.296270	0.016*	
C2	0.48116 (19)	0.37213 (18)	0.08897 (18)	0.0128 (5)	
H2A	0.490640	0.340303	0.028633	0.015*	
H2B	0.427627	0.320959	0.130269	0.015*	
C3	0.66953 (18)	0.53329 (18)	0.07806 (18)	0.0114 (5)	
H3A	0.731342	0.579620	0.112530	0.014*	
H3B	0.686222	0.507770	0.017146	0.014*	
C4	0.53229 (19)	0.5242 (2)	−0.01127 (19)	0.0136 (5)	
H4A	0.548519	0.492784	−0.067233	0.016*	
H4B	0.507300	0.564661	−0.041889	0.016*	
C5	0.4345 (2)	0.4943 (2)	0.14521 (19)	0.0149 (5)	
H5A	0.406948	0.534767	0.120457	0.018*	
H5B	0.385635	0.443460	0.190774	0.018*	
N1	0.52210 (16)	0.55474 (17)	0.20140 (16)	0.0134 (4)	
N2	0.45315 (15)	0.44554 (16)	0.05507 (16)	0.0120 (4)	
H2	0.391704	0.410798	0.014182	0.014*	
N3	0.61909 (16)	0.58570 (16)	0.04598 (16)	0.0124 (4)	
P1	0.59476 (5)	0.43083 (5)	0.16489 (5)	0.01082 (13)	
P2	0.666667	0.333333	0.41562 (8)	0.0119 (2)	
Ag1	0.666667	0.333333	0.22792 (2)	0.00986 (8)	
C6	0.34261 (19)	0.22240 (19)	0.32803 (19)	0.0118 (5)	
O3	0.3890 (2)	0.1866 (2)	0.28981 (18)	0.0361 (6)	
O6	0.32156 (19)	0.27601 (18)	0.27414 (18)	0.0295 (5)	
O7	0.31591 (19)	0.2064 (2)	0.41785 (18)	0.0316 (5)	
C10	0.666667	0.333333	0.8982 (3)	0.0112 (7)	
O2	0.60792 (17)	0.36433 (18)	0.89697 (18)	0.0279 (5)	

### (I) Atomic displacement parameters (Å^2^)

**Table:**

	*U*^11^	*U*^22^	*U*^33^	*U*^12^	*U*^13^	*U*^23^
C7	0.0127 (12)	0.0189 (13)	0.0139 (11)	0.0068 (11)	0.0001 (9)	0.0023 (10)
C8	0.0170 (13)	0.0140 (12)	0.0176 (12)	0.0096 (11)	−0.0013 (10)	−0.0017 (10)
C9	0.0171 (13)	0.0143 (12)	0.0137 (11)	0.0064 (10)	0.0015 (9)	0.0028 (9)
N6	0.0129 (11)	0.0176 (12)	0.0127 (10)	0.0071 (9)	0.0005 (8)	−0.0002 (9)
C1	0.0140 (12)	0.0174 (13)	0.0110 (10)	0.0096 (10)	−0.0006 (9)	−0.0007 (9)
C2	0.0131 (12)	0.0124 (12)	0.0136 (11)	0.0068 (10)	0.0005 (9)	−0.0007 (9)
C3	0.0092 (11)	0.0126 (11)	0.0136 (10)	0.0063 (10)	0.0004 (9)	0.0023 (9)
C4	0.0151 (12)	0.0156 (12)	0.0113 (10)	0.0086 (10)	0.0003 (9)	0.0011 (10)
C5	0.0145 (12)	0.0190 (13)	0.0144 (11)	0.0108 (11)	−0.0003 (9)	−0.0046 (9)
N1	0.0132 (10)	0.0153 (11)	0.0147 (10)	0.0094 (9)	−0.0015 (8)	−0.0021 (8)
N2	0.0111 (10)	0.0153 (11)	0.0112 (9)	0.0078 (9)	−0.0020 (8)	−0.0031 (8)
N3	0.0152 (10)	0.0109 (10)	0.0132 (9)	0.0081 (9)	0.0004 (8)	0.0013 (8)
P1	0.0116 (3)	0.0115 (3)	0.0113 (3)	0.0072 (3)	0.0004 (2)	0.0008 (2)
P2	0.0129 (3)	0.0129 (3)	0.0098 (4)	0.00647 (16)	0.000	0.000
Ag1	0.00977 (10)	0.00977 (10)	0.01003 (12)	0.00489 (5)	0.000	0.000
C6	0.0129 (11)	0.0140 (11)	0.0131 (10)	0.0102 (9)	0.0013 (8)	−0.0025 (8)
O3	0.0494 (14)	0.0499 (14)	0.0311 (11)	0.0413 (12)	0.0055 (10)	−0.0026 (10)
O6	0.0389 (14)	0.0341 (13)	0.0288 (12)	0.0283 (12)	0.0167 (10)	0.0141 (10)
O7	0.0349 (13)	0.0448 (15)	0.0234 (11)	0.0260 (12)	0.0044 (10)	0.0044 (10)
C10	0.0140 (11)	0.0140 (11)	0.0055 (15)	0.0070 (5)	0.000	0.000
O2	0.0252 (11)	0.0325 (12)	0.0327 (12)	0.0194 (10)	−0.0028 (9)	−0.0079 (9)

### (I) Geometric parameters (Å, º)

**Table:**

C7—H7A	0.9900	C3—H3B	0.9900
C7—H7B	0.9900	C3—N3	1.469 (3)
C7—N6	1.470 (3)	C3—P1	1.846 (3)
C7—P2	1.855 (3)	C4—H4A	0.9900
C8—H8A	0.9900	C4—H4B	0.9900
C8—H8B	0.9900	C4—N2	1.521 (3)
C8—N1	1.471 (3)	C4—N3	1.435 (3)
C8—N3	1.472 (3)	C5—H5A	0.9900
C9—H9A	0.9900	C5—H5B	0.9900
C9—H9B	0.9900	C5—N1	1.433 (3)
C9—N6^i^	1.463 (4)	C5—N2	1.521 (3)
C9—N6	1.468 (4)	N2—H2	1.0000
C1—H1A	0.9900	P1—Ag1	2.4699 (6)
C1—H1B	0.9900	P2—Ag1	2.4683 (11)
C1—N1	1.473 (3)	C6—O3	1.235 (3)
C1—P1	1.849 (3)	C6—O6	1.269 (3)
C2—H2A	0.9900	C6—O7	1.237 (3)
C2—H2B	0.9900	C10—O2^ii^	1.247 (2)
C2—N2	1.501 (3)	C10—O2	1.247 (2)
C2—P1	1.848 (3)	C10—O2^i^	1.247 (2)
C3—H3A	0.9900		
			
H7A—C7—H7B	107.9	N3—C4—N2	111.7 (2)
N6—C7—H7A	109.1	H5A—C5—H5B	107.9
N6—C7—H7B	109.1	N1—C5—H5A	109.3
N6—C7—P2	112.30 (18)	N1—C5—H5B	109.3
P2—C7—H7A	109.1	N1—C5—N2	111.8 (2)
P2—C7—H7B	109.1	N2—C5—H5A	109.3
H8A—C8—H8B	107.8	N2—C5—H5B	109.3
N1—C8—H8A	109.0	C8—N1—C1	111.6 (2)
N1—C8—H8B	109.0	C5—N1—C8	109.9 (2)
N1—C8—N3	112.9 (2)	C5—N1—C1	112.7 (2)
N3—C8—H8A	109.0	C2—N2—C4	111.21 (19)
N3—C8—H8B	109.0	C2—N2—C5	111.54 (19)
H9A—C9—H9B	107.6	C2—N2—H2	108.4
N6—C9—H9A	108.7	C4—N2—C5	108.8 (2)
N6^i^—C9—H9A	108.7	C4—N2—H2	108.4
N6^i^—C9—H9B	108.7	C5—N2—H2	108.4
N6—C9—H9B	108.7	C3—N3—C8	111.1 (2)
N6^i^—C9—N6	114.1 (2)	C4—N3—C8	110.8 (2)
C9^ii^—N6—C7	111.4 (2)	C4—N3—C3	111.9 (2)
C9—N6—C7	111.4 (2)	C1—P1—Ag1	120.57 (8)
C9^ii^—N6—C9	108.4 (2)	C2—P1—C1	97.77 (12)
H1A—C1—H1B	107.9	C2—P1—Ag1	120.17 (8)
N1—C1—H1A	109.2	C3—P1—C1	97.89 (12)
N1—C1—H1B	109.2	C3—P1—C2	98.11 (11)
N1—C1—P1	111.95 (16)	C3—P1—Ag1	117.54 (8)
P1—C1—H1A	109.2	C7^i^—P2—C7	97.50 (11)
P1—C1—H1B	109.2	C7^i^—P2—C7^ii^	97.50 (11)
H2A—C2—H2B	108.0	C7^ii^—P2—C7	97.50 (11)
N2—C2—H2A	109.4	C7^ii^—P2—Ag1	119.75 (8)
N2—C2—H2B	109.4	C7^i^—P2—Ag1	119.75 (8)
N2—C2—P1	111.12 (17)	C7—P2—Ag1	119.75 (8)
P1—C2—H2A	109.4	P1^i^—Ag1—P1	109.333 (15)
P1—C2—H2B	109.4	P1^ii^—Ag1—P1	109.332 (15)
H3A—C3—H3B	107.8	P1^ii^—Ag1—P1^i^	109.337 (15)
N3—C3—H3A	109.1	P2—Ag1—P1^ii^	109.608 (15)
N3—C3—H3B	109.1	P2—Ag1—P1	109.608 (15)
N3—C3—P1	112.47 (17)	P2—Ag1—P1^i^	109.608 (15)
P1—C3—H3A	109.1	O3—C6—O6	119.5 (2)
P1—C3—H3B	109.1	O3—C6—O7	121.0 (3)
H4A—C4—H4B	107.9	O7—C6—O6	119.5 (2)
N2—C4—H4A	109.3	O2^i^—C10—O2	119.982 (11)
N2—C4—H4B	109.3	O2^i^—C10—O2^ii^	119.983 (11)
N3—C4—H4A	109.3	O2^ii^—C10—O2	119.984 (11)
N3—C4—H4B	109.3		
			
N6—C7—P2—C7^i^	49.24 (17)	N2—C5—N1—C8	57.1 (3)
N6—C7—P2—C7^ii^	−49.39 (16)	N2—C5—N1—C1	−68.1 (3)
N6—C7—P2—Ag1	179.93 (15)	N3—C8—N1—C1	68.4 (3)
N6^i^—C9—N6—C7	67.4 (3)	N3—C8—N1—C5	−57.3 (3)
N6^i^—C9—N6—C9^ii^	−55.5 (3)	N3—C3—P1—C1	−49.28 (19)
N1—C8—N3—C3	−68.3 (3)	N3—C3—P1—C2	49.79 (19)
N1—C8—N3—C4	56.7 (3)	N3—C3—P1—Ag1	−179.92 (14)
N1—C1—P1—C2	−50.6 (2)	N3—C4—N2—C2	−68.5 (3)
N1—C1—P1—C3	48.8 (2)	N3—C4—N2—C5	54.7 (3)
N1—C1—P1—Ag1	177.40 (14)	P1—C1—N1—C8	−60.9 (2)
N1—C5—N2—C2	67.2 (3)	P1—C1—N1—C5	63.3 (2)
N1—C5—N2—C4	−55.8 (3)	P1—C2—N2—C4	60.7 (2)
N2—C2—P1—C1	50.14 (19)	P1—C2—N2—C5	−60.9 (2)
N2—C2—P1—C3	−49.03 (18)	P1—C3—N3—C8	61.3 (2)
N2—C2—P1—Ag1	−177.56 (12)	P1—C3—N3—C4	−63.1 (2)
N2—C4—N3—C8	−55.4 (3)	P2—C7—N6—C9^ii^	60.8 (3)
N2—C4—N3—C3	69.2 (3)	P2—C7—N6—C9	−60.4 (3)

Symmetry codes: (i) −*y*+1, *x*−*y*, *z*; (ii) −*x*+*y*+1, −*x*+1, *z*.

### (I) Hydrogen-bond geometry (Å, º)

**Table:**

*D*—H···*A*	*D*—H	H···*A*	*D*···*A*	*D*—H···*A*
N2—H2···O6^iii^	1.00	1.73	2.731 (3)	176

Symmetry code: (iii) −*x*+*y*+1/3, −*x*+2/3, *z*−1/3.

## References

[bb1] Armstrong, D., Kirk, S. M., Murphy, C., Guerriero, A., Peruzzini, M., Gonsalvi, L. & Phillips, A. D. (2018). *Inorg. Chem.* **57**, 6309–6323. 10.1021/acs.inorgchem.8b00227 29791143

[bb2] Barnes, P. A., O’Connor, M. F. & Stone, F. S. (1971). *J. Chem. Soc. A* pp. 3395.

[bb3] Bruker (2014). *APEX2* and *SAINT*. Bruker AXS Inc., Madison, Wisconsin, USA.

[bb4] Darensbourg, D. J., Decuir, T. J., Stafford, N. W., Robertson, J. B., Draper, J. D., Reibenspies, J. H., Kathó, A. & Joó, F. (1997). *Inorg. Chem.* **36**, 4218–4226.

[bb5] Dolomanov, O. V., Bourhis, L. J., Gildea, R. J., Howard, J. A. K. & Puschmann, H. (2009). *J. Appl. Cryst.* **42**, 339–341.

[bb6] Jimenez, J., Chakraborty, I., Rojas-Andrade, M. & Mascharak, P. K. (2017). *J. Inorg. Biochem.* **168**, 13–17. 10.1016/j.jinorgbio.2016.12.009 PMC5728992 27997857

[bb7] Kirillov, A. M., Smoleński, P., Guedes da Silva, M. F. C. & Pombeiro, A. J. (2007). Wiley Online Library.

[bb8] Kong, L.-Y., Zhang, Z.-H., Zhu, H.-F., Kawaguchi, H., Okamura, T.-A., Doi, M., Chu, Q., Sun, W.-Y. & Ueyama, N. (2005). *Angew. Chem. Int. Ed.* **44**, 4352–4355. 10.1002/anie.200500587 15962341

[bb9] Krause, L., Herbst-Irmer, R., Sheldrick, G. M. & Stalke, D. (2015). *J. Appl. Cryst.* **48**, 3–10. 10.1107/S1600576714022985 PMC4453166 26089746

[bb10] Krogstad, D. A., Ellis, G. S., Gunderson, A. K., Hammrich, A. J., Rudolf, J. W. & Halfen, J. A. (2007). *Polyhedron* **26**, 4093–4100.

[bb14] Parsons, S., Flack, H. D. & Wagner, T. (2013). *Acta Cryst.* B**69**, 249–259. 10.1107/S2052519213010014 PMC3661305 23719469

[bb11] Porchia, M., Benetollo, F., Refosco, F., Tisato, F., Marzano, C. & Gandin, V. (2009). *J. Inorg. Biochem.* **103**, 1644–1651. 10.1016/j.jinorgbio.2009.09.005 19822369

[bb12] Sheldrick, G. M. (2008). *Acta Cryst.* A**64**, 112–122. 10.1107/S0108767307043930 18156677

[bb13] Sheldrick, G. M. (2015). *Acta Cryst.* C**71**, 3–8.

